# Prenatal Exome Sequencing: When Does Diagnostic Yield Meet Clinical Utility?

**DOI:** 10.3390/genes17010037

**Published:** 2025-12-30

**Authors:** Alessia Carrer, Francesco Maria Crupano, Berardo Rinaldi, Giulietta Scuvera, Claudia Cesaretti, Valeria Nicotra, Silvana Gangi, Lorenzo Colombo, Gabriella Araimo, Matilde Tagliabue, Daniela Marchetti, Laura Pezzoli, Maria Garzo, Veronica Accurti, Grazia Volpe, Simona Boito, Palma Finelli, Monica Fumagalli, Maria Francesca Bedeschi, Maria Iascone, Nicola Persico, Federica Natacci

**Affiliations:** 1 Department of Health Sciences, University of Milan, 20122 Milan, Italy; 2 Medical Genetics Unit, Foundation IRCCS Ca’ Granda Ospedale Maggiore Policlinico, 20122 Milan, Italy; 3 Fetal Medicine and Surgery Service, Foundation IRCCS Ca’ Granda Ospedale Maggiore Policlinico, 20122 Milan, Italy; 4 Neonatal Intensive Care Unit, Foundation IRCCS Ca’ Granda Ospedale Maggiore Policlinico, 20122 Milan, Italy; 5 Department of Clinical Sciences and Community Health, University of Milan, 20122 Milan, Italy; 6 Medical Genetics Laboratory, ASST Papa Giovanni XXIII, 24127 Bergamo, Italylpezzoli@asst-pg23.it (L.P.);; 7 Medical Genetics Laboratory, Foundation IRCCS Ca’ Granda Ospedale Maggiore Policlinico, 20122 Milan, Italy; 8 Department of Medical Biotechnology and Translational Medicine, University of Milan, 20122 Milan, Italy

**Keywords:** prenatal exome sequencing, fetal anomalies, diagnostic yield, clinical utility, genotype-phenotype correlation

## Abstract

Background/Objectives: Prenatal Exome Sequencing (pES) has revolutionized prenatal diagnosis in fetuses with congenital anomalies. Although its performance is very promising, previous pES studies have mainly focused on diagnostic yield, often without considering the actual impact on ongoing pregnancies. In this study, we aim to (1) assess whether a prenatal molecular diagnosis can reliably predict the clinical features of the unborn child and (2) determine the gestational age (gw) at which ultrasound (US) findings are sufficient to support the pathogenicity of genetic variants detected by pES. Methods: We retrospectively selected 47 cases complicated by US anomalies that underwent Exome Sequencing (ES) and for which complete clinical assessment was available. A blinded reanalysis of ES data was performed, considering only prenatal features. Results: In our cohort, standard ES led to a molecular diagnosis in 43% of cases. The blinded reanalysis revealed that a complete or partial retrospective prenatal diagnosis was achievable in 95% of diagnosed cases. The mean gestational week at which US data would have supported molecular diagnosis was 22 + 5 weeks. The clinical follow-up confirmed a syndromic presentation in 21 out of 23 newborns and in all terminated pregnancies. Conclusions: Our study further confirms that pES is a valuable diagnostic tool for detecting genetic etiology in fetuses with congenital malformations. In most cases, pES results accurately predict the postnatal phenotype. However, the prenatal setting requires specific adjustments and precautions, and a negative pES result cannot be considered reassuring.

## 1. Introduction

Major congenital anomalies affect about 2.5% of pregnancies [1]. Ultrasound (US) screening is the standard for fetal anatomical evaluation. In approximately 10% of pregnancies, it raises an alarm that often leads to prenatal genetic investigations due to suspected syndromic conditions [2].

A definitive genetic diagnosis can significantly improve counseling by refining prognosis, management, and reproductive decision-making. However, because fetal phenotyping is limited, strong diagnostic hypotheses are rarely possible, and a genotype-first approach is often applied.

Chromosomal microarray (CMA) finds pathogenic copy number variants (CNVs) in 3% to 6.5% of structurally abnormal fetuses with normal karyotypes [3,4,5]. In 2019, two large prospective studies reported that prenatal Exome Sequencing (pES) provides an additional diagnostic yield of 8.5–10% [6,7]. Numerous smaller studies reported a diagnostic yield up to 80% in highly selected case series [8,9,10]. In 2022 a systematic review and meta-analysis found that pES can diagnose an additional 31% of cases with structural anomalies when karyotype and CMA are non-diagnostic [11]. A similar performance was confirmed also in later studies [12,13,14].

Despite these promising results, implementing pES in clinical practice faces challenges. Ongoing pregnancies limit the extent of fetal phenotyping via US and require short turnaround times (TATs). Whole-ES often identifies several candidate variants, complicating interpretation. Trio-based analysis (fetus and parents) is commonly used to narrow down called variants, but this approach can occasionally miss diagnoses. For instance, inherited dominant variants may be filtered out if passed from an undiagnosed parent in case of incomplete penetrance or parental somatic mosaicism.

Parental counseling must also address ethical concerns such as incidental findings, non-paternity, and individual inclination [5,15,16,17]. Complex or inconclusive results must be disclosed, as they can cause ambiguity and anxiety. To address these issues, recent guidelines from the International Society for Prenatal Diagnosis have outlined best practices for lab procedures, data analysis, and reporting [18].

Early pES studies mainly focused on diagnostic yield without considering the actual course of pregnancy [6,7,19]. More recent work has focused on clinical utility [20,21,22,23,24,25,26,27,28,29,30]. However, the robustness of ES in the context of ongoing pregnancies is still debated.

In this study, we aim to assess whether a prenatal molecular diagnosis can reliably predict the clinical features of the unborn child, given the known phenotypic variability. We also aim to clarify the gestational age (gw) at which US findings are sufficient to support the pathogenicity of genetic variants detected by pES.

To address these questions, we conducted a retrospective analysis of 47 cases with prenatal US anomalies. For each case, we collected prenatal and postnatal clinical data, as well as exome sequencing (ES) results obtained either after termination of pregnancy (TOP) or during the neonatal period. We then re-analyzed the ES data using only prenatal information and remained blinded to the final diagnosis.

## 2. Materials and Methods

The study was carried out in accordance with the principles laid down in the 2013 revision of the Declaration of Helsinki. Approval was obtained from the Ethical Committee and Scientific Board of the Fondazione IRCCS Ca’ Granda—Ospedale Maggiore Policlinico of Milan (No.6201-2022). All genetic investigations were performed in the diagnostic setting after obtaining written informed consent.

**Figure 1:**
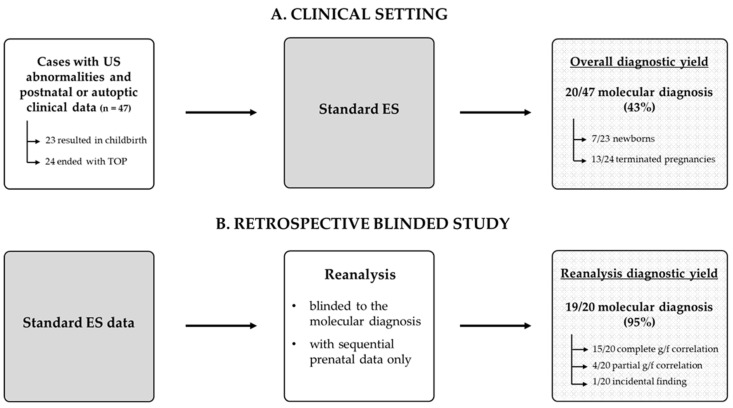
Clinical setting and retrospective study design. We retrospectively included 47 cases with prenatal abnormal US findings, of which 23 cases resulted in a live birth and 24 cases ended in termination of pregnancy (TOP). After using ES data for diagnostic purposes (**A**), we simulated pES by reanalyzing ES data (**B**) blinded to the molecular diagnosis, considering only prenatal findings at the gestational age at which they first appeared to evaluate genotype–phenotype (g/f) correlations.

The clinical setting and the retrospective study design are illustrated in Figure 1. We included all cases that met the following criteria: (1) ES performed either at birth or after TOP; (2) referral to our Fetal Medicine and Surgery Service during pregnancy due to abnormal US findings; and (3) non-diagnostic results from karyotype and/or CMA analysis.

We retrospectively included prenatal data of 47 fetuses belonging to 41 pregnancies. ES was carried out in the first month of life in 23/47 cases or after TOP in 24/47 cases. Standardized reports were used to collect prenatal anamnestic data, including family medical history, parental consanguinity, maternal age, conception method, twinning, and aneuploidy screening results. Fetal imaging, reviewed by a team specialized in prenatal US, was obtained for all cases. Fetal Magnetic Resonance imaging (MRI) was also collected when available. Neonatal clinical data were collected during follow-up. Fetal autopsy data, accompanied by MRI or Computed Tomography (CT) scans, were provided when requested for clinical purposes.

### 2.1. Cytogenetic Analysis

Conventional protocols were used to set up cultures and chromosome preparations. Placental, amniotic fluid or blood samples were cultured with standard techniques and chromosomal preparations were performed in situ. The applied banding technique was QFQ, using the MetaSystems software for karyotyping. The analyses were performed in accordance with the national Italian Society of Human Genetics (SIGU) guidelines 2014 [31], which are consistent with the European guidelines 2019 [32].

Genomic DNA from peripheral blood samples from probands and their parents was extracted using QIAsymphony DNA Kits (Qiagen, Hilden, Germany), according to the manufacturer’s instructions; genomic DNA from placenta/amniotic fluid was extracted using QIAcube (Qiagen, Hilden, Germany), both according to the manufacturer’s instructions. A total of 128 DNA samples were analyzed. Molecular karyotyping was performed through CMA using SurePrint G3 Human CGH Array Kit, 8x60K and 4x180K (Agilent Technologies, Santa Clara, CA, USA). Labeling and hybridization were performed according to the manufacturer’s protocol. Agilent Feature Extraction was used to quantify the fluorescence of the scanned images and Cytogenomics software v5.1 was used for data analysis. Copy number variant (CNV) call was performed using the ADAM-2 algorithm. Probe positions are referred to the Human Genome assembly GRCh37/hg19. The CNV classification was performed according to the guidelines suggested by the American College of Medical Genetics and Genomics (ACMG) [33]. Both the karyotype and the molecular karyotype were described in accordance with the international system for human cytogenomic nomenclature (ISCN) 2020 [34].

### 2.2. ES Analysis and Blinded Reanalysis

ES has been performed at the Medical Genetics Laboratory of ASST Papa Giovanni XXIII of Bergamo. The exonic regions and flanking splice junctions of the genome were captured using the Clinical Research Exome v.2 kit (Agilent Technologies, Santa Clara, CA, USA). Sequencing was performed on a NextSeq500 Illumina system (Illumina, San Diego, CA, USA) with 150 bp paired-end reads. Sequence reads were mapped to the reference human genome assembly (February 2009, GRCh37/hg19) and analyzed by the BWA enrichment version 2.0 pipeline and a second independent in-house pipeline [35].

The variant call file (VCF), including single-nucleotide polymorphisms and indels, was annotated by querying population frequencies databases and mutation databases, including the Genome Aggregation Database (http://gnomad.broadinstitute.org/) [36], ClinVar https://www.ncbi.nlm.nih.gov/clinvar/, accessed on 17 October 2022) [37] and Human Gene Mutation Database Professional (HGMD, Release 2017.4). Variants were classified based on ACMG guidelines [38]. The potential causative variants were subsequently confirmed by Sanger sequencing in the proband and parents using an independent DNA sample.

After using ES data for diagnostic purposes and returning reports to the families in the genetic counseling setting, ES data were reanalyzed for the blinded reanalysis considering only prenatal findings blind to postnatal or autoptic data (Figure 1). To simulate the progression of pregnancy, we interpreted ES data, matching them with prenatal clinical data only (US and MRI findings) at any gestational age of appearance. For each case, we assessed the stage of pregnancy in which US data would have been sufficient to support the real pathogenicity of the molecular data. The ES data filtering strategy was based on the virtual gene-panel “Fetal anomalies” (version 1.92) and “Fetal hydrops” (version 1.42) for a total of 1033 (high-evidence, green) genes, implemented by PanelApp [39], a publicly available database that proposes lists of genes with a high level of evidence of disease causation, which are suitable for inclusion in an automated pipeline for ES analysis. Written informed consent for genetic tests was obtained from the parents of the fetus or newborn.

## 3. Results

### 3.1. Family and Pregnancy Data

We evaluated 47 cases whose pregnancies were complicated by abnormal US findings (Supplementary Materials Table S1—Summary, and Table S2—Prenatal findings). 35 out of 47 (74%) cases were unrelated. Six cases (6/47) involved three pairs of affected fetuses in subsequent pregnancies (Cases 25–26, 31–32 and 35–36). The remaining six cases (6/47) were twin pregnancies (Cases 11–12, 13–14 and 28–29), in which both fetuses presented with US anomalies. In 23 out of 47 cases (49%), the pregnancy resulted in a live birth, while in 24 cases (51%), the pregnancy ended with TOP.

In Cases 22, 25–26, parents were consanguineous (3/47, 6%), being first and second cousins, respectively. All pregnancies were achieved without donor gametes, except for Case 15 conceived via oocyte donation. Among the fetuses, 26 out of 47 (55%) were females, and 21 out of 47 (45%) were males. Aneuploidy screening was performed in 30 out of 47 (64%) cases, resulting in low risk in 14 (47%), intermediate risk in 1 out of 30 (3%), and high risk in 15 (50%). The mean gestational week (gw) at first US findings was 19 + 2 gw.

The most recurrent US finding categories were multisystem anomalies (25/47, 53%), hydrops/lymphatic/effusion (6/47, 13%), central nervous system (CNS) anomalies (5/47, 11%), abdominal findings (3/47, 6%), and cardiac findings (3/47, 6%). Fetal MRI was performed in 9 out of 47 cases (19%), and four fetuses underwent prenatal surgery (three thoracoamniotic shunts and one fetoscopic endoluminal tracheal occlusion).

Overall, karyotype and/or CMA were performed in 42 of 47 cases. Prenatal invasive procedures (chorionic villus sampling or amniocentesis) were carried out in 39 fetuses (83%), while karyotype and/or CMA were performed postnatally in 3 additional newborns. All analyses yielded normal or non-diagnostic results; only small inherited CNVs were identified in Case 1 and Cases 28–29. No pathogenic genomic abnormalities were detected within the resolution limits of the cytogenetic techniques employed.

### 3.2. Postnatal/Postmortem ES Results

ES was performed as a trio-based analysis in 34 out of 47 cases, as quadruplex in 12 cases due to twin pregnancy or recurrence of fetal malformations (Cases 11–12, 13–14, 25–26, 28–29, 31–32, 35–36), and as duo in Case 15 because of oocyte donation.

A molecular diagnosis was reached in 20 out of 47 (43%) cases (Table 1), of which 7 were newborns (7/20, 35%) and 13 were terminated pregnancies (13/20, 65%). Case 21 was not included among the diagnosed cases, since ES data did not provide an explanation for the US findings but led to an incidental diagnosis of *MYH9*-related macrothrombocytopenia (OMIM #155100, ORPHA:182050), prenatally undetectable but confirmed in the newborn and his mother through a blood count.

11 diagnoses involved a dominant condition, related to eight de novo variants and two variants inherited from an affected parent (Cases 35 and 36); in Case 15, segregation of the identified *KMT2D* variant was confirmed only in the paternal allele due to oocyte donation. 8 of the 20 diagnoses were recessive conditions, of which 5 involved homozygous variants and 3 compound heterozygous variants. Only one molecular diagnosis involved an X-linked recessive variant inherited from the mother (*L1CAM*, case 45). Among the causative variants, eleven had been previously described in the literature, while the remaining were novel.

In our cohort, a molecular diagnosis was more likely when US findings were suggestive for skeletal dysplasia (1/1, 100%), followed by multisystem involvement (13/25, 52%), hydrops/lymphatic/effusion (3/6, 50%), renal alterations (1/2, 50%), and CNS anomalies (2/5, 40%). No pathogenic variants were identified in cases with isolated abdominal anomalies (0/3), cardiac anomalies (0/3) or intrauterine growth restriction (IUGR) (0/1).

### 3.3. Genotype–Phenotype Correlation with Postmortem/Postnatal Data

23 pregnancies resulted in a live birth (Cases 1 to 23). Subsequent follow-up clinical data were available for all newborns except for Case 13, totaling 452 months of follow-up (median 32 months, range 0–63) (Supplementary Materials Table S3—Follow-up data).

Based on clinical evolution, we confirmed all molecular diagnoses. Importantly, we were able to evaluate the natural history of 14 subjects with negative ES results. A syndromic clinical picture was confirmed in 21/23 infants, including 1 child (Case 20) who received a clinical diagnosis of Currarino syndrome (OMIM #176450, ORPHA:1552). The remaining 2 children demonstrated normal development. Prenatal US findings in this latter group were heterogeneous: Case 3 had no malformations but presented with oligo/anhydramnios, while Case 9 showed bilateral hydrothorax.

24 pregnancies resulted in TOP (Cases 24 to 47). Autopsy was performed in only 19/24 cases, as it was not possible in the remaining 5 due to early surgical TOP. Additionally, five MRI and nine CT scans were conducted after TOP.

In all cases except Case 41, for which the diagnosis remained partial even in routine diagnostic workup, postnatal or autopsy clinical data were essential for establishing a definitive diagnosis. In Case 6, X-rays at the age of four years confirmed *SLC26A2*-related recessive multiple epiphyseal dysplasia. In related Cases 25–26, autopsies identified hepatomegaly and erythroblastosis, supporting a diagnosis of *SEC23B*-related congenital dyserythropoietic anemia type II in both pregnancies.

In related Cases 31–32 and 35–36, autopsy findings further supported the diagnosis of two conditions rarely identified prenatally. Cases 31–32 has been previously reported [40]. In the first pregnancy, a 20 gw US detected a long philtrum, clinodactyly of the 5th finger, bilateral hydronephrosis, short femurs, and polyhydramnios. Autopsy confirmed these US findings and additionally revealed dysmorphic ears, a flat nose with anteverted nares, micro-retrognathia, and bilateral brachydactyly with 5th finger clinodactyly. In the subsequent pregnancy, US detected multiple anomalies, including increased prenasal thickness, nasal bone hypoplasia, micrognathia, bilateral pyelectasis, clinodactyly of the 5th finger, hepatomegaly, and shortened lower limbs. Fetal MRI detected enlarged subarachnoid spaces, mild ventriculomegaly, hypoplasia of the cerebellar vermis, hypoplasia of the corpus callosum, and dysmorphic features of the brainstem. Postmortem examination confirmed brachycephaly, flat glabella, anteverted nares, a long and deep philtrum, cleft palate, retrognathia, short limbs, bilateral 5th finger clinodactyly, incomplete lung lobulation, intestinal malrotation, bilateral hydronephrosis, and a bicornuate uterus. Although the two *PIGW* variants were initially classified as variants of unknown significance (VUSs), the prenatal phenotype and the segregation pattern across subsequent pregnancies suggested a likely causative role. Postnatal findings further supported their pathogenic classification.

Related Cases 35–36 have also been previously reported [41]. The first pregnancy was complicated by bilateral renal agenesis and anhydramnios, prompting the couple to opt for TOP at 19 gw. Autopsy confirmed bilateral renal agenesis and revealed additional anomalies, including agenesis of the corpus callosum, macrocephaly with dolichocephaly, hypertelorism, a depressed nasal ridge, posteriorly rotated and low-set ears, bilateral lung hypoplasia, uterine agenesis, and mild clubfeet. The CT scan also identified multiple rib anomalies. In the following pregnancy, US at 21 gw identified mild ventriculomegaly, hypertelorism and unilateral cleft lip and palate. Fetal MRI confirmed ventriculomegaly and identified mildly incomplete opercularization, reduced hemispheric thickness, altered anteroposterior pons diameter, and increased interocular distance. The couple chose to terminate the pregnancy at 22 gw. Autopsy findings included lateral ventricle enlargement, hypertelorism, depressed glabella, nasal ridge depression, and confirmed the left cleft lip and palate. Postmortem MRI further detected altered sulcation, hypoplastic falx cerebri, thalami and fornix asymmetry, and hypoplasia of the cerebellum and bulbar olives. CT imaging revealed multiple bilateral rib anomalies. A subsequent diagnosis of Basal cell nevus syndrome (BCNS) in the mother allowed for the identification of a maternal PTCH1 variant in both fetuses. Despite some variation, both fetuses exhibited features overlapping with postnatal BCNS, and prenatal findings alone could retrospectively have supported the diagnosis.

### 3.4. Blinded Study: Genotype–Phenotype Correlation with Prenatal Data Only

Of the 20 diagnosed cases in the standard clinical setting, we reviewed the genotype–phenotype correlations to assess whether and when prenatal clinical data alone (US and MRI findings) might have supported the molecular diagnosis if the pregnancies had been ongoing.

**Figure 2:**
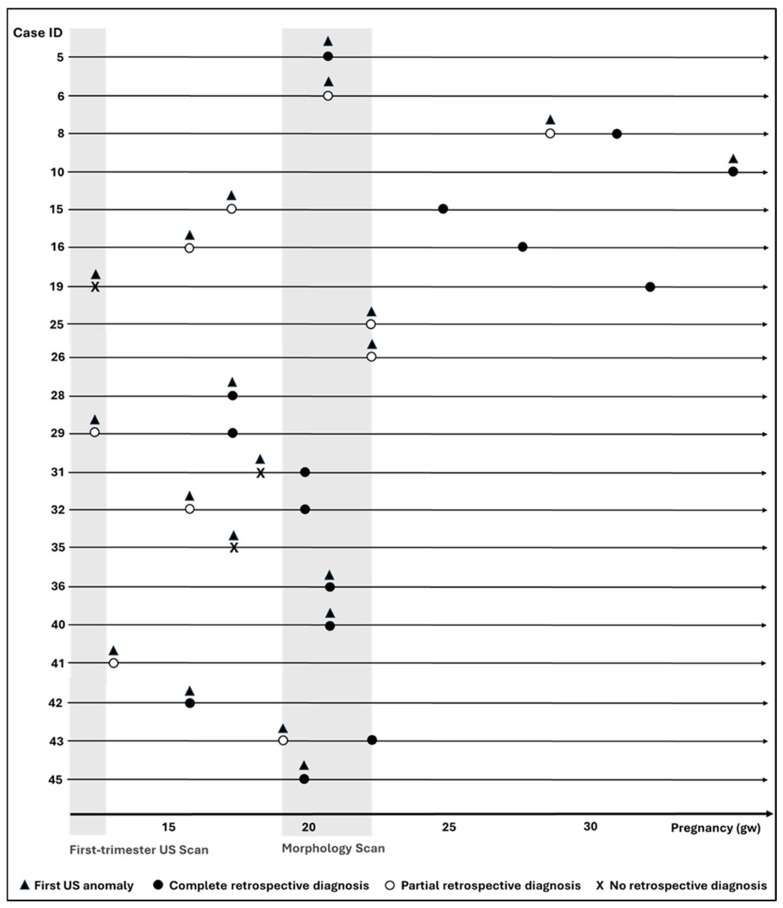
Time of first US findings, partial and complete retrospective diagnosis of the blinded study. The image illustrates the timeline spanning from the 10th to the 40th gestational week (gw). Each line corresponds to a single case, whose ID is indicated by numbers at the beginning of each line. First US anomalies are marked with full black triangles. Complete retrospective diagnoses are represented by full black circles, while partial retrospective diagnoses are indicated by white circles. A “X” indicates no retrospective diagnosis. Grey shading highlights the gestational periods for the first- and second-trimester US scans.

As detailed in Figure 2 (and Supplementary Materials Table S4—Blinded study), a retrospective prenatal diagnosis was achievable in 19/20 (95%) diagnosed cases. The remaining one is Case 35, since for these subsequent pregnancies (Cases 35–36), a retrospective diagnosis based solely on prenatal data was possible only for the second pregnancy (Case 36), while for the first one, US findings were nonspecific.

The genotype–phenotype correlation was complete for 15/20 (75%) cases. In 4/20 (20%), the genotype–phenotype correlation was only partial, as not all US findings matched the known clinical features of the underlying condition (Cases 6, 25–26, 41). In particular, in Case 6, the observed shortening of the long bones by US did not correspond with the phenotype of recessive *SLC26A2*-related multiple epiphyseal dysplasia (OMIM #226900, ORPHA:93307). In subsequent pregnancies 25–26, the diagnosis of *SEC23B*-related congenital dyserythropoietic anemia type II explained polyhydramnios and hydrops in both fetuses but not the cerebellar and skeletal anomalies. Finally, in Case 41, US performed at 13gw revealed micro-retrognathia, bilateral cleft palate, persistent flexion of the upper limbs, hypoplasia of the right hand, and aplasia of the left hand and feet. ES identified the likely pathogenic variant c.463A>G in *MYH3*, which would not explain the acral defects. However, the pregnancy was terminated before 15 gw, preventing the autopsy and direct confirmation of limb defects.

Among the 15 cases with a complete genotype–phenotype correlation, the match was possible from the first US abnormalities in 7/20 diagnosed cases (35%), whereas the evolution of US findings allowed a reclassification from partial to complete in the other 6 cases (Cases 8, 15, 16, 29, 32 and 43) at 31, 25, 28, 17, 20, and 22 gw, respectively. From this blinded reassessment, the mean of the earliest gw when US data supported the molecular diagnosis was 22 + 5 gw (range 16–36 gw). The mean gw for requesting ES, based on NHS Rapid Prenatal Exome Sequencing Inclusion Criteria [42], would be 21 + 4 gw (range 13–36 gw).

## 4. Discussion

Prenatal US represents the cornerstone of fetal anomaly detection. However, its ability to predict postnatal outcome is inherently limited. Structural imaging alone cannot fully capture functional impairments, particularly those affecting neurodevelopment, which may not manifest as detectable anomalies during pregnancy. In this context, genetic testing provides a critical complementary tool by uncovering the molecular basis of fetal conditions and refining prognostic assessment.

Traditionally, prenatal genetic evaluation has relied on conventional karyotyping and CMA, which allow the detection of aneuploidies and pathogenic CNVs. However, they leave a substantial proportion of cases unexplained. In recent years, the first studies on pES have demonstrated its significant contribution to increasing diagnostic yield [6,7,11]. More recent research has focused on its clinical utility in ongoing pregnancies [22,23,29,30]. However, only a limited number of studies have included postnatal or postmortem follow-up data.

To address this challenge, we retrospectively reanalyzed genetic and phenotypic data from 47 cases complicated by fetal anomalies. We selected cases with thorough and consistent clinical assessments. These included postnatal follow-up or fetal autopsy. This approach allowed us to validate molecular diagnoses and assess the clinical utility of pES in ongoing pregnancies.

Overall, in our cohort, ES provided a molecular diagnosis in 43% of pregnancies complicated by US abnormalities with normal karyotype and CMA. This result is consistent with previous reports [11,12,13,14].

The blinded reanalysis showed that a retrospective prenatal diagnosis would have been possible in most diagnosed cases (19/20, 95%). Genotype–phenotype correlation was complete in 15 out of 20 cases (75%) and partial in the remaining 4 (25%). Therefore, in our cohort, albeit small, if ES had been conducted prenatally, it would not have led to incorrect diagnoses. On the other hand, pES would have yielded ambiguous or inconclusive results in 6/47 (13%) cases. These included four partial diagnoses (Cases 6, 25–26, 41), one with no retrospective diagnosis (Case 35) and one incidental finding unrelated to US anomalies (Case 21). Evidence of ambiguous or partial pES results has also been reported by others [5,14,43,44]. This confirms that the possibility of incidental or partial diagnoses should always be addressed during pre-test counseling. Ethical concerns may also arise in this context. They include potential misinterpretation, incomplete phenotypic characterization, or the possibility of double diagnoses. These factors could further complicate counseling and decision-making.

Previous studies reported pES TAT or mean gw at referral [8,12,23,28]. However, the gestational timing at which a definitive molecular diagnosis could be associated with fetal anomalies has not been fully investigated. In our study the mean gestational age at which US data would have supported a molecular diagnosis was 22 + 5 gw. Based on current NHS criteria [35], pES would have been requested, on average, at 21 + 4 gw (Figure 2). This timing likely reflects the standard schedule of US checkpoints in uncomplicated pregnancies. It is in line with observations from other studies [20,23,26].

A diagnosis established at 21–22 gw may have relevant real-world implications, as it occurs close to the legal limits for pregnancy termination in our country. This timing could influence clinical management and parental counseling. For example, it may result in limited windows for genetic testing, variant interpretation, and, if needed, additional investigations. It may also constrain the period available for fully informed parental decision-making. Beyond ethical considerations, these findings also highlight issues related to the organization of prenatal care and the timing of US. It should be noted, however, that earlier gestational assessments may make genotype–phenotype correlations more challenging.

Our second aim was to correlate ES results with clinical follow up data from both diagnosed and undiagnosed newborns or fetuses. Among diagnosed cases, the phenotypic evolution of newborns during follow-up was consistent with the natural history of the identified genetic conditions. Except for Cases 8 and 10, in which prenatal or neonatal clinical data strongly suggested Noonan syndrome, the diagnoses in other cases notably benefited from the genotype-driven approach followed by reverse phenotyping. In pregnancies resulted in TOP, autopsy findings were consistent with ES results in all 13 diagnosed cases.

In molecularly undiagnosed cases (27/47, 57%), clinical follow-up confirmed a syndromic presentation in 14 out of 16 infants (88%). Only one child (Case 18) received a clinical diagnosis (Currarino syndrome—OMIM #176450, ORPHA:1552). Only 2/16 undiagnosed children with prenatal US abnormalities showed normal development (13%). These data suggest that a negative pES result should not be considered reassuring, and fetal prognosis should not overlook US-detected anomalies. Similarly, in all pregnancy resulted in TOP with negative ES results (11/24, 46%), autopsy findings indicated a highly plausible, albeit unknown, multisystemic or syndromic condition. This result may nevertheless be affected by the bias that, in our center, ES was applied either at birth or after TOP in cases presenting with the most severe phenotypes. In any case, for pregnancies complicated by US abnormalities, the clinical utility of pES is notably more consistent and reliable for positive results than for negative ones.

As evaluated in previous studies and currently adopted by NHS [6,35], our initial pES filtering strategy focused on minimizing the detection of VUS. We used a virtual gene panel adapted from PanelApp, including only genes known to be associated with fetal anomalies [39]. Related Cases 28–29 represent a limitation. The causative gene (*WDR81*) was not included in the panel at the time of first analysis and was identified only upon later reanalysis.

Dominant conditions accounted for most diagnoses (11/20), including eight de novo variants and two inherited variants from an affected parent (Cases 35–36). Since the filtering strategy may rely on inheritance status, dominant inherited variants may be missed unless parental phenotype is known. Therefore, it is essential to collect a detailed family history before performing pES, keeping in mind that the prenatal and postnatal manifestations of a condition may not be immediately correlated. Indeed, in related Cases 35–36, the pathogenic variant in PTCH1 was identified only in the second pregnancy, as the maternal diagnosis of Basal Cell Nevus Syndrome (BCNS) was still unknown during the first pregnancy. The causative *PTCH1* variant might have been suspected also in the first pregnancy if the dominant filter strategy had not been applied. Conversely, including inherited variants in the analysis may increase the detection of VUS, incidental findings, and secondary findings, which in turn may prolong TATs and complicate clinical management.

Finally, the study’s strengths include data collection at a single referral center for prenatal diagnosis, ensuring consistency and reliability. Availability of postnatal follow-up and autopsy data provided a comprehensive understanding of both clinical outcomes and natural history of the diagnosed conditions. This allowed us to confirm molecular diagnoses and evaluate the effectiveness of pES in a real-world clinical setting.

However, our study had also strong limitations. The retrospective nature of this case series, combined with the small sample size, may have led to selection bias and reduces the strength and generalizability of the conclusions. Moreover, ES was performed only after negative or inconclusive karyotype/CMA results and was primarily aimed at single-nucleotide variant detection. Although clinical ES can also enable the identification of CNVs and potentially increase diagnostic yield—particularly for CNVs below the resolution of the CMA used—at the time these analyses were conducted, a robust and validated bioinformatic pipeline for CNV detection from WES data was not yet available in our diagnostic workflow. More recently, the implementation of prenatal genome sequencing may further increase diagnostic yield by allowing the detection of small genomic rearrangements as well as intronic and other non-coding variants that are not captured by exome-based approaches.

## 5. Conclusions

This study further demonstrates that pES is a valuable diagnostic tool for detecting the genetic etiology of congenital malformations in fetuses. In our cohort, ES provided a molecular diagnosis in 43% of cases. The blinded reanalysis showed that, even in prenatal settings with limited access to detailed fetal phenotypes, a complete genotype–phenotype correlation was achievable in most diagnosed cases (75%), with a mean gestational age of 22 + 5 gw. Moreover, thanks to our detailed clinical follow-up, we were able to confirm in newborn or after TOP the molecular diagnosis virtually obtained by pES. At the same time, we underline how negative pES cannot be interpreted as reassuring, since a syndromic presentation was typically confirmed at autopsy or in the postnatal period.

We evaluated that a filtering approach utilizing virtual gene panels and guided by inheritance patterns proved to be an effective method for identifying variants in morbid genes linked to prenatal onset, while simultaneously reducing the detection of VUSs. Our findings support the integration of pES into routine clinical practice, especially when combined with detailed US examinations.

Besides detailed family anamnesis, an extensive and detailed sonography report helps to direct the pES data for a final diagnosis. This holds especially true when new US anomalies appear during the course of pregnancy and should therefore be shared with the laboratory doing the exome. To further enhance the clinical utility of pES, additional routine US assessments may be warranted to provide the timely detection of anomalies. Furthermore, to ensure an accurate correlation between US-defined fetal anomalies and genetic data, it is essential that centers requesting pES have access to an expert fetal medicine unit, as well as both clinical and laboratory medical genetics services, all integrated within a multidisciplinary team.

Although the study is small and retrospective, it provides a good ground for following studies.

## Figures and Tables

**Table 1:** Pregnancy data and ES results of diagnosed cases (*n* = 20).

Case ID	GW of First US Finding	Category	Prenatal Anomalies	ES Results
5	21	Hydrops/Lymphatic/Effusion	Bilateral hydrothorax	*EPHB4* (NM_004444.5):c.2216G>A, p.(Arg739Gln) *het dn*
6	21	Skeletal	Long bone shortening	*SLC26A2* (NM_000112.4):c.835C>T, p.(Arg279Trp) *hom*
8	29	Hydrops/Lymphatic/Effusion	Macrocephaly, cerebrospinal fluid space enlargement, polyhydramnios, hepatomegaly	*NRAS* (NM_002524.5):c.34G>A, p.(Gly12Ser) *het dn*
10	36	Hydrops/Lymphatic/Effusion	Bilateral hydrothorax, polyhydramnios, short long bones, hypoplastic kidneys	*PTPN11:* c.923A>C, p.(Asn308Thr) *het dn*
15	17	Renal	Hypoplastic left pelvic kidney, SUA, polyhydramnios	*KMT2D* (NM_002834.5):c.14710C>T, p.(Arg4904ter) *het*
16	16	Multisystem	Fallot tetralogy, IUGR	*GNPAT* (NM_014236.4):c.1280-2A>G/p.Arg335ter *ch*
19	12	Multisystem	IUGR, brachydactyly, interventricular septum hypertrophy	*SMAD4* (NM_005359.6): c.1499T>C, p.(Ile500Thr) *het dn*
25	22	Multisystem	Polyhydramnios, hydrops, short long bones	*SEC23B* (NM_006363.6):c.716A>G, p.(Asp239Gly) *hom*
26	22	Multisystem	Polyhydramnios, short long bones, cerebellar hypoplasia	*SEC23B* (NM_006363.6):c.716A>G, p.(Asp239Gly) *hom*
28	17	Multisystem	Oligohydramnios, ventriculomegaly, cerebellum not detected, micrognathia	*WDR81* (NM_001163809.2):c.1403G>A/c.4160del, p.(Arg468His/Phe1387SerfsTer13) *ch*
29	17	Multisystem	Oligohydramnios, ventriculomegaly, cerebellum not detected, micrognathia, Fallot tetralogy	*WDR81* (NM_001163809.2):c.1403G>A/c.4160del p.(Arg468His/Phe1387SerfsTer13) *ch*
31	18	Multisystem	Polyhydramnios, bilateral hydronephrosis, short long bones, clinodactyly	*PIGW* (NM_001346754.2):c.827T>C, p.(Leu276Pro) *hom*
32	16	Multisystem	Bilateral pyelectasis, short long bones, micrognathia, cerebellar and corpus callosum hypoplasia	*PIGW* (NM_001346754.2):c.827T>C, p.(Leu276Pro) *hom*
35	17	Multisystem	Renal agenesis, anhydramnios	*PTCH1* (NM_000264.5):c.648_651dup, p.(Gln218fs*) *het mat*
36	21	Multisystem	Cleft lip and palate, ventriculomegaly, hypertelorism	*PTCH1* (NM_000264.5):c.648_651dup, p.(Gln218fs) *het mat*
40	20	Multisystem	Diaphragmatic hernia, hypoplastic corpus callosum, low-set ears, clenched hands	*SMARCA4* (NM_003072.5):c.3557C>T, p.(Ala1186Val) *het dn*
41	13	Multisystem	Micrognathia, cleft palate, persistent flexion of the arms, hand and foot agenesis	*MYH3* (NM_002470.4):c.463A>G, p.(Ile155Val) *het dn*
42	16	CNS	Semilobar holoprosencephaly	*NIPBL* (NM_133433.4):c.6801G>T, p.(Met2267Ile) *het dn*
43	19	Multisystem	Hydrothorax, cardiac hypertrophy, small and low-set ears, hypertelorism, hypospadias	*NIPBL* (NM_133433.4):c.5482C>G, p.(Arg1828Gly) *het dn*
45	20	CNS	Ventriculomegaly, hypoplastic corpus callosum	*L1CAM* (NM_001278116.2):c.3774G>C, p.(Ter1258Tyrext*264) *hem mat*

Legend: *hom:* homozigosity; *ch*: compound heterozygosity; CNS: central nervous system; *dn*: de novo; ES: exome sequencing; GW: gestational week; *hem*: hemizygosity; *het*: heterozygosity; IUGR: intrauterine growth restriction; *mat*: maternal; SUA: Single Umbilical Artery; US: Ultrasounds imaging.

## Data Availability

Data available on request due to restrictions.

## Supplementary Materials

The following supporting information can be downloaded at: https://www.mdpi.com/article/10.3390/genes17010037/s1, Table S1: Detailed prenatal findings; Table S2: Family and pregnancy data; Table S3: clinical follow-up data of newborns; Table S4: Genotype-phenotype correlation with prenatal data only.

## Abbreviations

The following abbreviations are used in this manuscript:

pES	Prenatal Exome Sequencing
US	Ultrasound
ES	Exome Sequencing
CMA	Chromosomal Microarray
TATs	Turnaround Times
TOP	Termination Of Pregnancy
MRI	Magnetic Resonance imaging
CT	Computed Tomography
CNV	Copy Number Variant
VCF	Variant Call File
gw	Gestational Week
CNS	Central Nervous System
IUGR	Intrauterine Growth Restriction
